# APM_GUI: analyzing particle movement on the cell membrane and determining confinement

**DOI:** 10.1186/2046-1682-5-4

**Published:** 2012-02-20

**Authors:** Silvia A Menchón, Mauricio G Martín, Carlos G Dotti

**Affiliations:** 1 Department of Molecular and Developmental Genetics, VIB Center for the Biology of Disease and Center for Human Genetics, KULeuven, Campus Gasthuisberg, Herestraat 49 - bus 602, 3000 Leuven, Belgium; 2 IFEG-CONICET and FaMAF, Universidad Nacional de Córdoba, Córdoba, Argentina; 3 Centro de Biología Molecular Severo Ochoa, CSIC-UAM, Campus Universidad Autónoma de Madrid, Madrid, Spain

## Abstract

**Background:**

Single-particle tracking is a powerful tool for tracking individual particles with high precision. It provides useful information that allows the study of diffusion properties as well as the dynamics of movement. Changes in particle movement behavior, such as transitions between Brownian motion and temporary confinement, can reveal interesting biophysical interactions. Although useful applications exist to determine the paths of individual particles, only a few software implementations are available to analyze these data, and these implementations are generally not user-friendly and do not have a graphical interface,.

**Results:**

Here, we present APM_GUI (Analyzing Particle Movement), which is a MatLab-implemented application with a Graphical User Interface. This user-friendly application detects confined movement considering non-random confinement when a particle remains in a region longer than a Brownian diffusant would remain. In addition, APM_GUI exports the results, which allows users to analyze this information using software that they are familiar with.

**Conclusions:**

APM_GUI provides an open-source tool that quantifies diffusion coefficients and determines whether trajectories have non-random confinements. It also offers a simple and user-friendly tool that can be used by individuals without programming skills.

## Background

Informatics tools have become essential for biologists because they allow the analysis of a vast amount of data obtained using newly developed techniques and electronic devices. Many informatics tools are now available, which makes it possible to analyze various processes, such as the linear movement of particles, and to quantify fluorescence using easy-to-use software. However, more complex biological processes, such as the multi-directional, evanescent and at times frantic movement of proteins diffusing along the lateral plane of membranes, require sophisticated algorithms that run on complex software distributions. These applications sometimes require a thorough knowledge of programming, which makes their use difficult for biologists. The aim of this work is to provide a user-friendly, accessible and open-source tool for the analysis of data from two-dimensional single-particle tracking (SPT) experiments.

SPT is a useful technique for the study of the dynamics and movement of individual sub-micrometer-sized particles. SPT is frequently used to study the movement of receptors on the cell surface [1]. The development of quantum dots (Qdots) and the improved sensitivity of fluorescence microscopy allow the localization of each particle with a high resolution and precision for long periods of time [2]. SPT occurs in two important and distinct stages: first, the dots (representing the particles' positions) are localized and marked, and second, the dots are tracked over time [3]. The trajectories for each particle are obtained by trace reconnection. Although many sources of noise are present throughout the process, (e.g., the blinking of Qdots, which generates dark states), some algorithms are able to label and track the marked particles. The most commonly used implementations for Qdots are MTT (a set of MatLab scripts [4]), Particle Tracker (a ImageJ plug-in [5]) and Imaris Track (Bitplane's software [6]). However, it is difficult to find a user-friendly application with a graphical interface that can analyze the dynamic properties of trajectories, such as variations in protein diffusion coefficients.

Here, an implementation for MatLab with a graphical user interface (GUI) is presented that allows the analysis of lateral diffusion properties using data obtained by SPT in two dimensions. In particular, it determines whether a particle has free Brownian motion or non-random confinement based on how long the particle stays in a given region [7,8]. When a particle trajectory shows regions with confined movement, our implementation determines where it occurs, the sizes of the confined regions, the duration of the time that a particle stays in each of these regions and the characteristic diffusion coefficients. Although this application runs on MatLab, (and saves all of the data in a workspace for MatLab users), all generated data are also exported in *.dat *files that can be opened with any software and subjected to statistical analyses, which allows the users to analyze and plot this information using software that they are familiar with.

## Implementation

**Figure 1:**
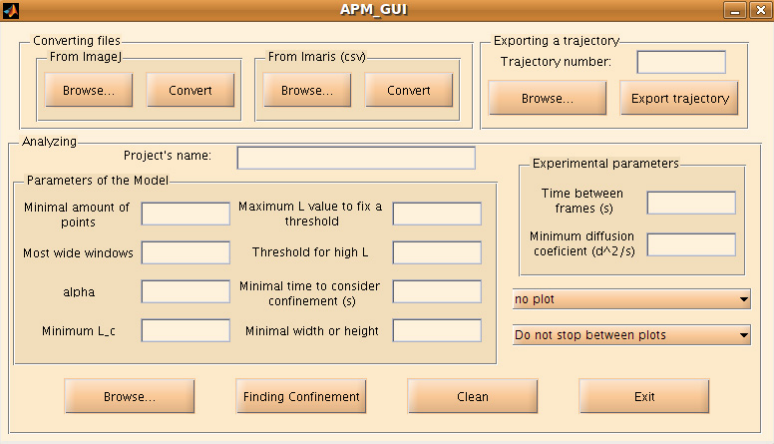
**APM_GUI window**. The APM_GUI window contains three panels: *Converting files, Analyzing *and *Exporting a trajectory*. Information about process status is displayed in the MatLab *Command Window*.

APM_GUI is a complete GUI tool created using MatLab language and GUIDE. The MatLab development is platform-independent. The installation consists of unpacking the compressed folder that contains a collection of MatLab scripts and functions to the desired location and adding this location to the MatLab path. It is possible to add directories to the MatLab path by selecting *Set Path *at *File *in the menu bar (and then *Add folder*). If this folder is not added permanently to the MatLab path, these steps must be performed each time MatLab is started. No manual compilation is necessary because the graphical interface appears after APM_GUI is written in the MatLab Command Windows and is composed of three panels: *Converting files, Analyzing *and *Exporting a trajectory*, as shown in Figure 1. APM_GUI is distributed using a short user manual (see additional file 1). The open-source nature of the software allows expert users to adjust each script according to their own needs and edit the routines running on the GUI.

### Data import

Data obtained through SPT experiments must be converted to a MatLab format. The panel *Converting *calls the scripts to convert files from Imaris and ImageJ. An extra script is provided to convert files from MTT (see additional file 1). If Imaris is used to obtain the trajectories, its output has to be saved as *.csv *file. Then, using the *"Browse" *button in the panel *"From Imaris (csv)"*, the *.csv *files are selected, and their conversion starts after the *"Convert" *button is pressed in the *"From Imaris (csv)" *panel. If the Particle Tracker of ImageJ is used to obtain the trajectories, its output has to be saved as *.txt *file. Then, the procedure is the same as in the case of data obtained through Imaris, but the *"Browse" *and *"Convert" *buttons in the panel *"From ImageJ" *are used instead.

### An algorithm based on the Simson/Saxton approach

To detect the confinement zones on each trajectory, we implement an algorithm reported by Simson and co-workers [7], which is based on Saxton's work [9] with the addition of a few modifications related to the threshold definition. This algorithm is a well-established method to determine confinement and has been used in recent publications [4,10-12]. On a particle trajectory, confinement zones are defined as the places where the particle remains for a duration of time that is considerably longer than a Brownian diffusant would stay. Thus, we first need to know how long a particle with Brownian motion would stay in a given region. This factor has already been studied by Saxton [9], who determined the following mathematical expression to describe the probability *ψ *that a particle, with Brownian motion and constant diffusion coefficient *D*, will stay in a region of radius *R *for a period of time *t*:

(1) $$\text{log}\left(\psi \right)=0.2048-2.5117Dt/R^{2}.$$

The coefficients in Eq. (1) can be obtained from the series expansion of the analytical solution (see Appendix A in Ref. [9]).

We are interested in regions where a particle with Brownian motion has a low probability of staying for a period of time *t*, i.e., regions characterized by the lowest values of *ψ*. Therefore, it is possible to define a confinement index, *L*, by [7]:

(2) $$L=\left\{\begin{array}{cc} -\text{log}\left(\psi \right)-1 & \psi \leq 0.1,\\ 0 & \psi >0.1. \end{array}\right)$$

The index *L *is always a positive number, and non-random confinement zones are correlated with its highest values. To determine the confinement index for an experimental trajectory, which can be represented as a sequence of points, we proceed by breaking it down into shorter segments. Thus, each point on the trajectory is taken as a starting point for a series of segments with variable length, from four to *S*_*m*_. Then, to calculate the index *L*, we define *R *as the largest displacement from the starting point and *t *as the duration of the segment, (if the segment has *n *frames, *t *= (*n - *1)Δ, where Δ is the time between frames). We also take the diffusion coefficient in Eq. (1), *D*, for each trajectory from the highest instantaneous diffusion coefficient. Because each point on the trajectory is included in many segments, the final value for *L*ata given point is the average over all segments that it belongs to. Regions for non-random confinement are associated with the highest values of *L*. Thus, a period of confined diffusion is defined by the positions where *L *increases above a critical threshold *L*_*c *_for a duration of time longer than a critical time *t*_*c*_. A confinement zone position is defined by the center, which is the average position of all points with *L *≥ *L*_*c*_, and the distance from the center to the furthest point into the confinement zone. Because the diffusion coefficient of a particle can change along the trajectory, we define *L*_*c *_similarly to Meilhac *et al*. [8]. In our algorithm, *L*_*c *_= *α *⟨*L*⟩, where ⟨.⟩ represents the average over all the points along the trajectory. Because Brownian diffusants do not have non-random confined movement, we define a minimum possible value for *L*_*c*_. However, to prevent mistakes in defining the size of the confinement zone, we fix the value of the threshold *L*_*c *_if *L *has very high values. This value is fixed to ensure well-defined confinement zone sizes in the cases of experimental trajectories that are confined most of the time or in cases in which there is a very large difference between the outer and inner diffusion coefficients. Thus, for each trajectory, the threshold *L*_*c *_is generally defined as *α*⟨*L*⟩. However, if *α*⟨*L*⟩ is less than the minimum possible value, *L*_*c *_is defined as the minimum possible value, and if *L *has very large values, *L*_*c *_assumes another predefined value (see the section "The parameters").

### Diffusion coefficients

The diffusion coefficients were quantified by analyzing the mean square displacement (MSD). The MSD (*ρ*) is the average value for the square of the distance between the initial and final positions of a particle for all time lags. Using all available displacements of a given duration *n*Δ, we have:

(3) $$\rho_{n}=\frac{1}{N-n}\overset{N-n}{\underset{i=1}{\sum }}{\left({\vec{r}}_{n+i}-{\vec{r}}_{i}\right)}^{2},\;n=1,...,N-1,$$

where *N *is the total number of points in the trajectory. For small *n*, the MSD values are well averaged. However, longer time lags have greater uncertainty in the MSD value. Diffusion coefficient values can be obtained from the evaluation of a plot of the MSD vs. time. However, the localization uncertainty and finite camera exposure introduce an *offset *to the MSD curve. Michalet considered all of these effects (see Section III Ref. [13]) and proposed an algorithm to determine the optimal number of points to be considered when fitting the MSD curve, (see Section V Ref. [13]).

The characteristic diffusion coefficient of a given region is calculated as described by Michalet [13] using a sub-trajectory containing only the points within the region under consideration. Instantaneous diffusion coefficients are calculated by computing an MSD curve for each sub-trajectory of duration 10Δ. Although the uncertainty in the diffusion coefficient can be relatively large for such a short sub-trajectory, instantaneous diffusion coefficients are useful in determining slow and fast diffusion regimes with durations equal to or greater than 10Δ (the used time window).

### The parameters

The parameters *S*_*m*_, *t*_*c *_and all of the parameters involved in the definition of *L*_*c *_must be optimized. A random walk can temporarily mimic confinement. Then, the *L*-profile would not be a horizontal line, even for a Brownian diffusant. However, the peaks for a random walk with mimicked confinement would be lower and narrower than those for non-random confinement. Then, the minimum value for *L*_*c *_has to ensure that the *L*-profile for any Brownian diffusant would be below it (or above it, but only for durations shorter than *t*_*c*_). This parameter has to be introduced in the "Minimum L_c" text box. However, to prevent mistakes in the determination of confinement zone sizes, if *L *has a greater value than the parameter introduced in the "Maximum L value to fix a threshold" text box, then the threshold *L*_*c *_takes the value that has been introduced in the "Threshold for high L" text box. These two parameters cannot affect most trajectories; they are defined only for specific cases and obviously do not affect any trajectory when the parameter entered in the "Maximum L value to fix a threshold" text box is sufficiently high. The value of *S*_*m *_changes the *L*-profile. If *S*_*m *_is too low, the *L*-profile presents many peaks. Increasing the maximum segment size *S*_*m *_smooths the *L*-profile. However, if *S*_*m *_is too large, the *L*-profile is very smooth, and information about confinement is lost. This loss is caused by taking the average over very large segments of the trajectory. Then, the value of *S*_*m *_must be sufficiently high to suppress the mimicked confinement of Brownian motion. However, it cannot be too high because the *L*-profile resulting from non-random confinement may also be suppressed. The optimization of the time *t*_*c *_depends on *S*_*m *_and *L*_*c*_. However, depending on the biological sample, it can sometimes be estimated from previous knowledge. For a more detailed description about how to optimize the parameter *S*_*m*_, see Ref. [7], as we kept the same notation.

### Inputs and outputs

The files to analyze can be selected using the browser for the folder dialog box that appears after the "Browse" button in the panel "Analyzing" is pressed. For the analysis, APM_GUI uses the files that contain trajectory information that has been generated by the functions in the panel "Converting files", (see section Data import). When the files to analyze have been selected, and the required parameters have been given, the analysis will start after the "Finding confinement" button in the panel "Analyzing" is pressed.

**Figure 2:**
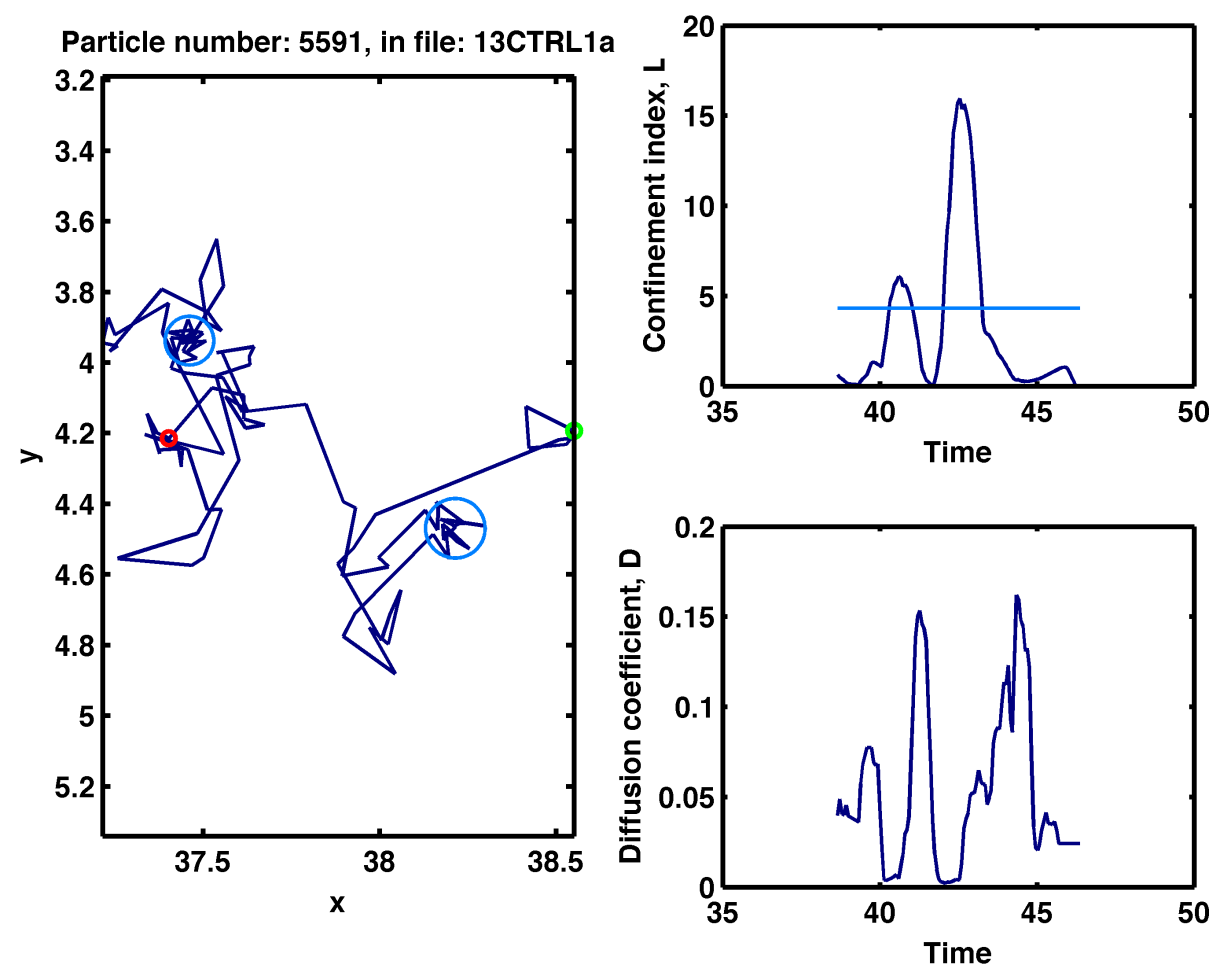
**Screen shot of a plot showing an AMPA receptor trajectory with confined regions**. A sample trajectory was registered for a single AMPA receptor in live hippocampal neurons under basal conditions (left panel). The green and red circles represent the first and last trajectory points, respectively. The confinement zones (light blue circles) correspond to regions in which the confinement index is above the critical threshold level, *L*_*c*_. The confinement index is shown in the upper-right panel, and the critical threshold, *L*_*c*_, is indicated by the light-blue line. The lower-right panel shows the instantaneous diffusion coefficient. In general, confinement zones associated with the movement of the AMPA receptor are correlated with lower values of the instantaneous diffusion coefficient. This trajectory corresponds to trajectory number 5591 in the file *13CTRL1a*. The distance and time units were *μm *and *s.*

The information from the analysis is saved in a .*mat *file. Users familiar with MatLab can then work on the project (performing statistical interpretation) after the analysis is complete. Information related to trajectories that do not have confinement zones is saved in the vectors with names containing "*nonConf*". Information for trajectories with confinement zones is divided into three groups: inside (including data about confinement zones characterized by a diffusion coefficient greater than the value that has been introduced in the "Minimum diffusion coefficient (d^2/s)" text box); immobile (including information about confinement zones characterized by a diffusion coefficient less than the value that has been introduced in the "Minimum diffusion coefficient (d^2/s)" text box); and outside (characterizing the trajectories outside confinement zones). All relevant data are also available in four files with a *.dat *extension: one with general information and three representing the inside, immobile and outside groups, as explained above. These files often have small sizes and are easily transported, which allows the statistical analyses to be performed using the user's preferred computer and/or software. Other options available in the panel "Analyzing" are referred to as plotting options. All trajectories, trajectories with non-confinement zones or trajectories with confinement zones can be selected for plotting. Confinement zones are represented by light and dark blue circles, which indicate mobile and immobile particles, respectively. The small green and red circles indicate the first and last points of the trajectory, respectively. The file name and the trajectory number are indicated at the top of the plot. An example of a plot is shown in Figure 2. For a detailed description, see the additional file 1.

### The panel: Exporting a trajectory

Using the panel "Exporting a trajectory", it is possible to export the spatial coordinates, the confinement index *L *and the instantaneous diffusion coefficient over time as a *.dat *file. In this case, the number of the selected trajectory has to be entered, and the file that contains it has to be selected using the browser for the folder dialog box that appears after the "Browse" button is pressed in the "Exporting a trajectory" panel. The parameters required in the empty boxes in the panel "Analyzing" also have to be entered. After the "Export trajectory" button is pressed, a new *.dat *file is created and saved in the same folder where the selected file is located.

## Results and Discussion

### Testing Simson/Saxton approach with simulated data

To test our implementation and analyze its power of detection, we used simulated random walks. Particle trajectories were generated by using a jump size *ℓ* and choosing a random direction for each jump. After 100 jumps, the particle position was recorded. Each simulated trajectory consisted of 100 frames (i.e., the particle position was recorded 100 times), and for each setting (i.e., for each *ℓ*), 1000 particles were simulated. The fixed jump length was determined by $\ell =\sqrt{4Ddt}$, where *D *was the diffusion coefficient applied and *dt *was the time interval of a basic simulation step. To simulate the confined motion, we assumed that the particles were entrapped in circular domains of radius *r*_*d*_. The exit from the domain was restricted by rigid walls. To test the effects of different noise levels, we displaced every trajectory point of a simulated trajectory by a random distance that was generated based on Gaussian white noise with a standard deviation of *σ*_*n*_. Because we also tested our implementation with experimental data obtained from AMPA receptors (see next sub-section), we used a similar setting for the simulations. Then, *dt *= 0.067*/*100 *s *and for the analysis, *S*_*m*_, *α *and *t*_*c *_were selected as 25, 0.5 and 0.67*s *(10 frames), respectively. The minimal value for *L*_*c *_was 4.3, and if *L *≥ 100, *L*_*c *_took the value of 19.

First, we analyzed a set of pure random trajectories, considering eight different diffusion coefficients between 0.01 *μm*^2^/*s *and 0.1 *μm*^2^/*s*. In all cases without noise, the false positive rate was less than 2.5%. However, this false positive rate decreased when noise was added. Simulations with noise were performed using standard deviations between 0.01 *μm *and 0.06 *μm*. In particular, all of the cases tested had a false positive rate of less than 1% if *σ*_*n *_≥ 0.05 *μm*.

**Figure 3:**
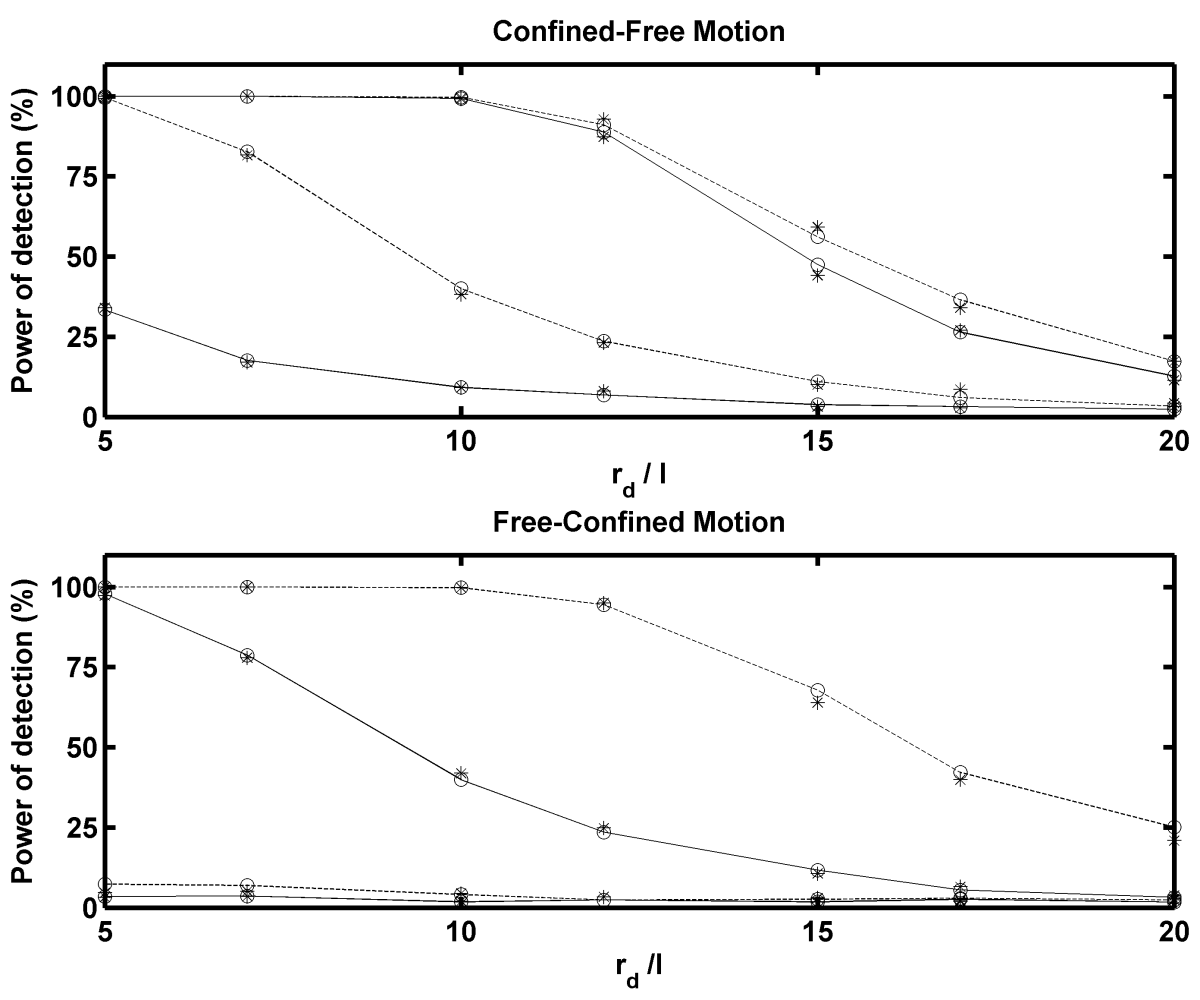
**Power of detection without noise**. The power of detection vs. the ratio *r*_*d*_/*ℓ* is plotted for *D *= 0.05 *μm*^2^/*s *(open circles) and *D *= 0.1 *μm*^2^/*s *(asterisk). The results for the confined-free motion scheme are shown in the upper panel and those for the free-confined motion scheme are presented in the lower panel. As expected, the power of detection increases considerably as the total number of frames, *M*, increases. In both panels, the lower solid and dashed lines represent the results for *M *= 11 and *M *= 15, respectively. The upper solid and dashed lines are used to express the results for *M *= 25 and *M *= 50, respectively.

**Figure 4:**
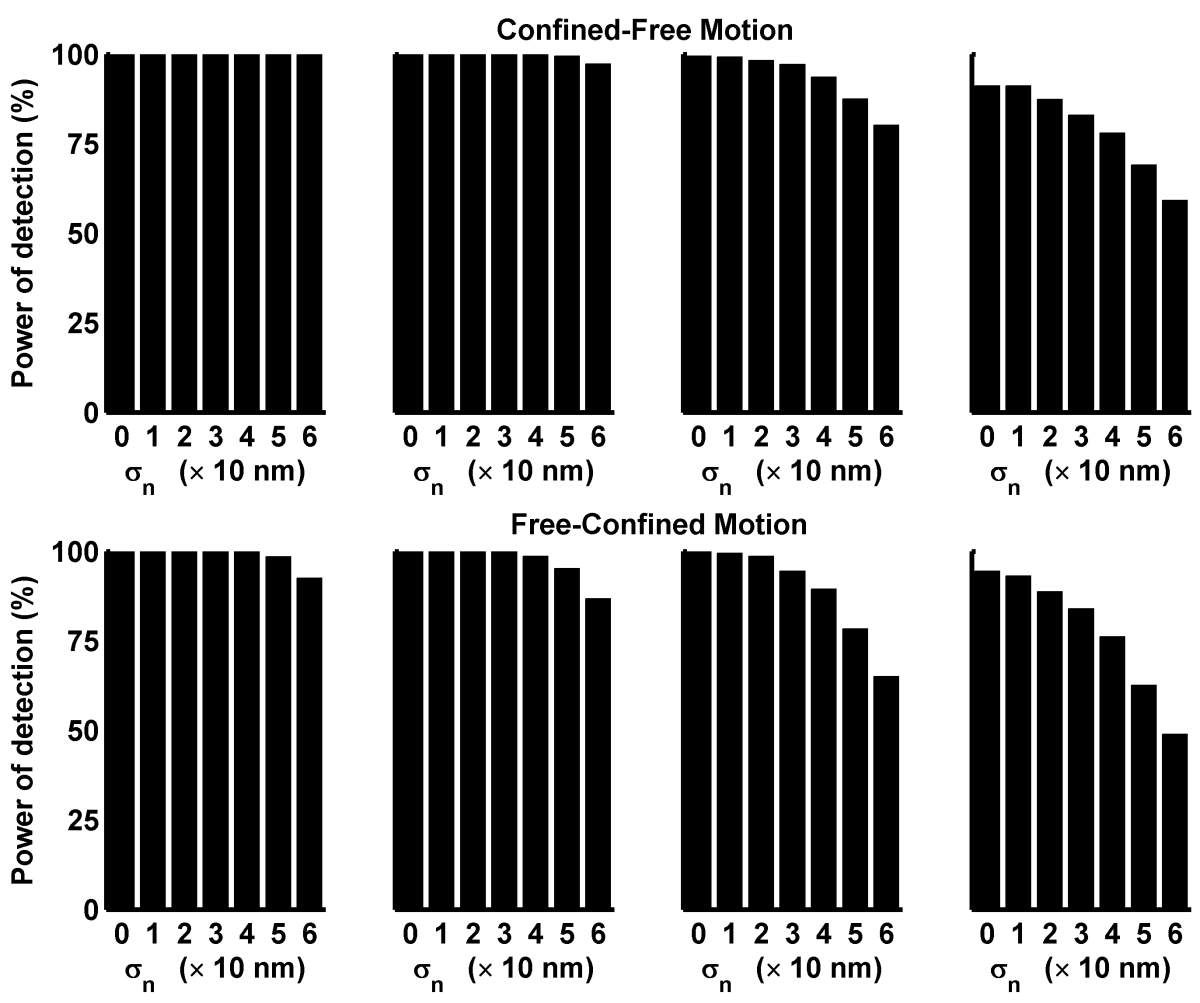
**Power of detection considering different noise levels**. Changes in the power of detection in response to different noise levels are shown for *D *= 0.05 *μm*^2^/*s, M *= 25, (A), and *M *= 50, (B). Gaussian white noise with a standard deviation of *σ*_*n *_was assumed. The values of *σ*_*n *_are indicated in the abscissa using a scale of 10 nm. In both panels (A and B), the first column corresponds to the ratio *r*_*d*_/*ℓ* = 5; the second to *r*_*d*_/*ℓ* = 7; the third to *r*_*d*_/*ℓ* = 10 and the fourth to *r*_*d*_/*ℓ* = 12.

To determine the power of detection of our implementation, we simulated trajectories with confinement zones. Two different types of mixed trajectories were generated. First, we considered that a particle started at the center of a confinement zone with a rigid wall and that the wall vanished after *M *frames. In other words, the particle was allowed only to jump inside the confinement zone for *M *frames, and afterward, it had a free Brownian motion without being confined. Second, we assumed that a particle with free Brownian motion entered a confinement zone through its edge at frame number 100 - *M *and remained in the confinement region until the last frame (100). In this case, the rigid wall appeared at the moment that the particle was entrapped. We called these schemes Confined-Free Motion and Free-Confined Motion, respectively. In Figure 3, the power of detection vs. *r*_*d*_/*ℓ* is plotted for different *M-*values for both types of mixed trajectories, without considering noise. As expected, better detection was provided with a smaller ratio *r*_*d*_/*ℓ* and a higher *M *(because the particles reached the boundary of the confinement zone more often). According to our settings, it is not possible to detect confinement if *M *≤ 10 and the last *S*_*m *_positions (25 points) are not averaged well. Therefore, the power of detection for free-confined motion is much lower than that for confined-free motion. The simulations were performed for *D *= 0.05 *μm*^2^/*s *(open circles) and *D *= 0.1 *μm*^2^/*s *(asterisk). When noise was added, the power of detection changed, as shown in Figure 4. Although Figure 4 shows the results for *D *= 0.05 *μm*^2^/*s*, similar results were obtained for *D *= 0.1 *μm*^2^/*s*. Considering that *ℓ* = 0.012 *μm *for *D *= 0.05 *μm*^2^/*s*, Figure 4 shows that the power of detection of our implementation is not strongly affected by noise levels that are similar to *ℓ*.

### Testing the Simson/Saxton approach with experimental data

We also tested our application by studying the diffusion of AMPA receptors on the cell membrane of hippocampal neurons maintained for 10-14 days *in vitro *under basal conditions of activity and after the addition of the excitatory neurotransmitter glutamate. For a detailed description of the material and methods, see additional file 2. We evaluated the data from five different neuronal cultures. The trajectories of single molecules were obtained by tracking with the Imaris program. Only trajectories containing at least 50 points were considered for the analysis; *S*_*m*_*, α *and *t*_*c *_were selected as 25, 0.5 and 0.67*s *(10 frames), respectively. The minimal value for *L*_*c *_was 4.3, and if *L *≥ 100, then *L*_*c *_assumed the value of 19.

**Figure 5:**
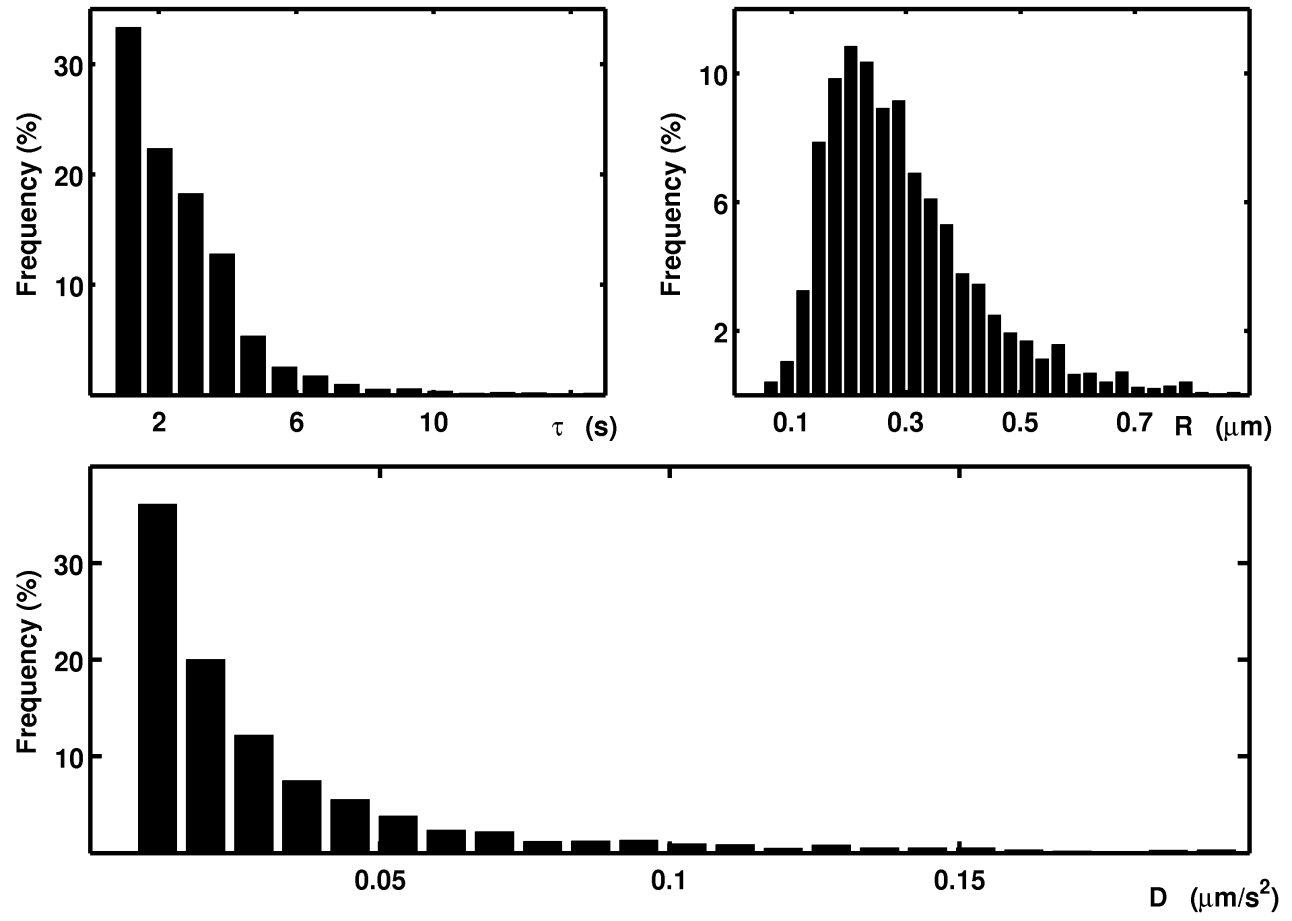
**Frequency histograms of the durations, sizes and diffusion coefficients of the confined events for a single culture**. Analysis for AMPA receptors in living hippocampal neurons after bath application of glutamate. The results are normalized to the total number of mobile confined zones.

In Figure 5, the histograms for the diffusion coefficients inside confined regions, the durations of confinement and the radii of the confined zones for a culture after the addition of the excitatory neurotransmitter glutamate are shown. The particles were considered to be immobile in a given region if the median of their instantaneous diffusion coefficients in that region was below 0.0075 *μm*^2^/*s*. The median was used because the diffusion coefficients for each culture had a long-tail distribution. Trajectories with confined zones were split into confined and non-confined regions by APM_GUI. For each type of region, a diffusion coefficient was assigned for computing the MSD curve. Non-confined regions were referred to as regions with free Brownian motion. Under basal conditions, the diffusion coefficient values for the mobile particles were significantly reduced in the confined zones, 0.0176 *μm*^2^/*s*, compared with free Brownian motion, 0.0732 *μm*^2^/*s *(*p *< 0.001, t-test, five cultures). After the application of glutamate, the confined and free diffusion coefficients for the AMPA receptors significantly increased: 0.0208 *μm*^2^/*s *(*p *< 0.05, t-test, 5 cultures) and 0.1097 *μm*^2^/*s *(*p *< 0.05, t-test, five cultures), respectively, compared with the basal conditions. This result suggests that the activation of hippocampal neurons with glutamate increases AMPA receptor diffusion. Significant variations were not observed in the size of the confined zones, the duration of permanence within the confined zones or the proportion of immobile particles in the confined zones. These results are consistent with previous studies [14-16].

## Conclusions

In conclusion, our application provides a robust tool to study confined movement and diffusion dynamics on the cell membrane. Although some parameters depend on the experimental equipment, many others have to be optimized according to the instructions given in the section "The parameters". This application is programmed in MatLab and has a GUI that allows it to be used even without any programming knowledge. However, expert users can modify the scripts according to their needs.

In general, the trajectories obtained by SPT are analyzed by fitting the MSD vs. time plot or by using a maximum likelihood estimator [17]. Both approaches give reliable diffusion coefficient values when they are used properly [13,17]. However, to identify standard types of motion, such as pure diffusion, anomalous diffusion, directed motion or confined movement, a specific algorithm should be provided. The test presented here is an algorithm based on the Simson/Saxton approach. This test has as its null hypothesis, *the particle has a pure random walk*, and as its alternative hypothesis, *the trajectory has confinement zones*. Thus, our implementation tests whether a trajectory corresponds to a pure random walk or if it has confinement zones. This type of algorithm is generally used to analyze biological data, when trapping is caused not only by the particles encountering a more viscous region but also by some type of barrier, such as a membrane skeleton fence [18]. Therefore, confined motion may result, for instance, from cytoskeletal corrals or from restrictions to motion that can be imposed by lipid domains [1]. Our implementation determines the confinement zones and characteristic diffusion coefficients very well. Although the power of detection decreases when the confinement region is at the end of the trajectory, a high power of detection can be obtained with relatively short trajectories, even in the presence of noise.

However, if a particle has a pure random walk, and if we wish to test if its diffusion coefficient changes along the trajectory, the Simson/Saxton approach would fail. In this case, an approach such as that presented by Montiel and co-authors should be used [17]. The Montiel test *always *assumes pure random walks, and the question behind it is: *are there changes in the diffusion coefficient values? *A statistical analysis of Montiel's test is presented in Ref. [17]. Although it is possible to obtain a high power of detection for diffusion coefficients differing by one order of magnitude, for smaller differences, considerably long trajectories are needed, which can be a limitation for biological samples on the cell membrane (because of endocytosis).

APM_GUI is based on the Simson/Saxton approach, and it is an open-source application that is publicly available directly from its home page and it is also included in the additional file 1. It also allows an easy analysis of all results because of the generation of files with a universal format and relative small size, which can be imported by any software for statistical analyses and plotting.

## Availability and requirements

**Project name**: AMP_GUI

**Project home page**: http://www.bits.vib.be/ under *BioInfo at VIB - Developed at VIB*.

**Operating system(s)**: platform-independent.

**Other requirements**: MatLab.

**License**: APM_GUI is distributed under the terms of the GNU General Public License, as published by the Free Software Foundation, version 3.

**Further information**: a short manual with a user's guide and installation instructions is available with the code source, which is also included in the additional file 1.

## List of abbreviations

The abbreviations used throughout the article are SPT: Single Particle Tracking; Qdots: Quantum dots; GUI: Graphical User Interface; MSD: Mean Square Displacement.

## Competing interests

The authors declare that they have no competing interests.

## Authors' contributions

SAM designed and developed the software. MGM contributed to the evaluation of the software regarding the biological data management and aided in its design. CGD initiated and supervised the project. Each of the authors contributed to the drafting of the manuscript and have read and approved its final version.

## Supplementary Material

Additional file 1 **Source code of APM_GUI**. The file should be extracted using a suitable program (e.g. Winzip, 7-Zip or File-roller). The extracted folder Scripts_APM_GUI should be placed in MatLab's path. Then, the application can be started by typing APM_GUI in MatLab's Command Window. The folder Examples has a few trial files for the software, and the file instructions_and_installation provides a short manual. Click here for file

Additional file 2 **Materials and Methods**. This file has a description of the materials and methods for the biological samples that were used to test our software. Hippocampal neurons from 18- day- old rat embryos were cultured until 10-14 DIV on glass coverslips, according to the Banker technique [19] density of (~ 6000 neurons/*cm*^2^). The protocol described in Ref. [20] was used to label GluR2-containing AMPARs. Click here for file
