# Supplementary material for: APM_GUI: analyzing particle movement on the cell membrane and determining confinement
Source: BMC Biophys. 2012 Feb 20;5:4. doi: 10.1186/2046-1682-5-4 (PMC3337278; doi:10.1186/2046-1682-5-4)
Supplement: Additional file 1 — Source code of APM_GUI. The file should be extracted using a suitable program (e.g. Winzip, 7-Zip or File-roller). The extracted folder Scripts_APM_GUI should be placed in MatLab's path. Then, the application can be started by typing APM_GUI in MatLab's Command Window. The folder Examples has a few trial files for the software, and the file instructions_and_installation provides a short manual. [file 2046-1682-5-4-S1.ZIP › Source_code_of_APM_GUI/Instructions_and_installation.pdf]

Instructions for running the MatLab implementation presented in "APM_GUI: analyzing particle movement on the cell membrane and determining confinement"

Silvia A. Menchón[1,*], Mauricio G. Martín[1,2], Carlos G. Dotti[1,2,*]
1 Department of Molecular and Developmental Genetics, VIB Center for the Biology of Disease and Center for Human Genetics, KULeuven, Campus Gasthuisberg, Herestraat 49 bus 602, 3000 Leuven, Belgium
2 Centro de Biología Molecular Severo Ochoa, CSIC-UAM, Madrid, Spain
∗ E-mail: SilviaAdriana.Menchon@cme.vib-kuleuven.be (SAM) and Carlos.Dotti@cme.vib-kuleuven.be (CGD)

Notice of Copyright

This implementation is a free software; you can redistribute it and/or modify it under the terms of the GNU General Public License as published by the Free Software Foundation, Version 3.
See *http://www.gnu.org/licenses/*.

The user acknowledges that the software is distributed "as is" without warranty and that neither the authors nor any distributor is liable for damage, loss of data or any other inconvenience caused by the use of the software.

This application is distributed free of charge for academic and non-commercial use. The copyright headers and names of the original authors in all program files and in this documentation must not be altered or removed. The paper "APM_GUI: analyzing particle movement on the cell membrane and determining confinement" by Menchón *et al.* must be cited in any publication that uses data generated or processed using this implementation or any proprietary extension of it.

A short introduction to MatLab

When you start MatLab, you will see a screen similar to the one shown in Fig. 1. At the top, you can see your `Current Directory`, (in the picture, it is the root directory on Linux). On the left, you can see all the folders and files that are in the `Current Directory`. The `Command Window` is in the middle. In this window, you input function and script names to be run on MatLab. Finally, the `Workspace` and `Command History` are on the right. If you do not see all of these windows, you can check their names on `Desktop` at the top of the MatLab window in the menu bar.

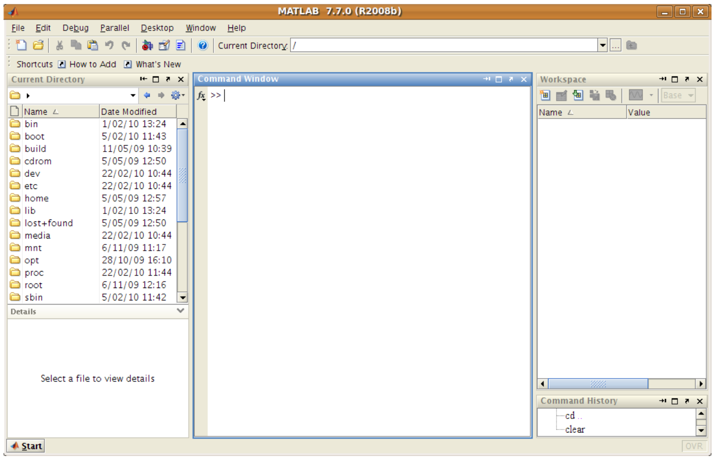

Figure 1. The MatLab window.

`Command History` window shows all of the commands that you have been using and `Workspace` windows shows all the variables that are currently available, (the `Workspace` in Fig. 1 is empty). Commands

should be written in the Command Window. A short list of the most commonly used commands is given in Table 1. To change the Current Directory, you can use the command cd writing on the Command Window: cd /home/username/foldername, as shown in Fig. 2a, or you can use the Browse for Folder dialog box by clicking on the icon that is shown in Fig. 2b.

Command	What does it do?
clear	It removes all variables from the Workspace
ls	It shows all of the files and folders in the Current Directory
cd ..	It changes the Current Directory to the directory above it.
cd *foldername*	If the Current Directory includes the directory *foldername*, this directory becomes the new Current Directory.
save *filename*	It stores all workspace variables in a binary format in the current directory in a file named *filename.mat*
load *filename*	It loads all of the variables from the MAT-file *filename.mat*, if it exists and returns an error if it does not exist.

Table 1. A few MatLab commands.

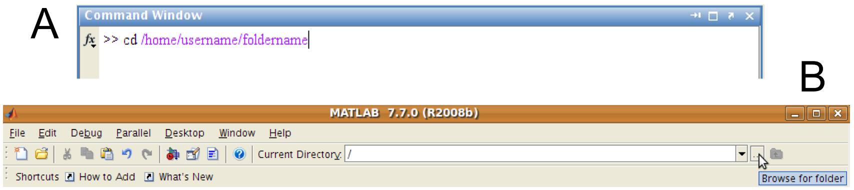

Figure 2. Changing the Current Directory.

Installation

The implementation runs on MatLab and has been tested for version 7.7.0.471 R2008b for Windows and Linux 32-bit. However, it should run in older and newer versions. The implementation consists of a collection of MatLab scripts and functions that are located in the code directory called *Scripts_APM_GUI*, and you have to save this folder in your local computer. **It is necessary to add this code directory to the MatLab path!** One easy way to do this is to run MatLab, click *File* the menu bar, select *Set Path*, followed by *Add folder* and finally you can add the newly created directory by choosing the folder using the common *Browse for Folder* dialog box, as shown in Fig. 3. These three steps are not needed when you save this path for use in future MatLab sessions. **When saving is not possible, you have to perform these steps each time you run MatLab**. You run APM_GUI on MatLab by writing APM_GUI on the MatLab Command Window and by pressing Enter.

Converting files

The application was developed with the assumption that the data are obtained using Imaris software or the Particle Tracker function of ImageJ. However, an additional script to convert files obtained with MTT is provided. We also describe the general structure of the .mat files that are used by APM_GUI.

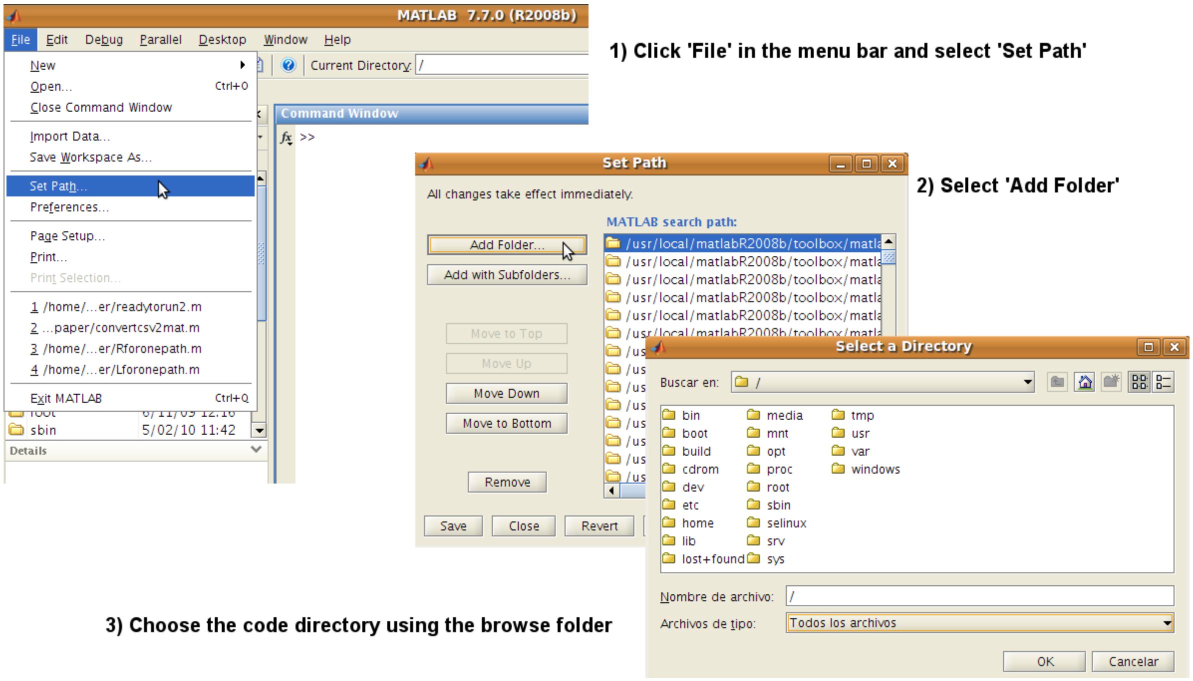

Figure 3. Adding a folder to the MatLab path.

Then, if other software has been used to obtain the trajectories, expert users can make their own scripts for the conversion.

1. **Using Imaris.** You have to export the data from the Imaris software in a .csv file, (select Statistics in the menu bar, then Detailed; choose specific values: position and save the export as comma-separated values). You have to choose a filename without spaces or forbidden characters, (*http://en.wikipedia.org/wiki/Filename*). If you open one of these .csv files with a text editor, you will see something similar to:

```
Detailed
====================
Position X,Position Y,Position Z,Unit,Category,Collection,Time,Parent,ID,
2.863,14.230,0.001,um,Spot,Position,1,1000000000,0,
2.851,14.245,0.001,um,Spot,Position,2,1000000000,11,
2.858,14.251,0.001,um,Spot,Position,3,1000000000,21,
```

   The procedure to convert this file to a MatLab format is the following: run APM_GUI on MatLab. After the graphical interface appears, you can select the files to convert using the browser for the folder dialog box that appears after pressing the “Browse” button in the panel “From Imaris”. You can select more than one file using the keys Ctrl and Shift, as usual. Once your files to convert have been selected, you press the “Convert” button in the panel “From Imaris”. Information about the conversion status is displayed on MatLab Command Window. New .mat files will be generated and saved in the same folder where the .csv files are located. This application has been tested on Imaris version 6.2.

2. **Using ImageJ.** After running Particle Tracker on ImageJ, a .txt file has to be saved by clicking full report; filenames cannot include forbidden characters, (you can see the reserved characters at *http://en.wikipedia.org/wiki/Filename*). If you open one of these .txt files with a text editor, you would see something similar to:

   ```
   % Configuration:
   %         Kernel radius: 3
   %         Cutoff radius: 3.0
   %         Percentile : 0.1
   %         Displacement : 10.0
   %         Linkrange : 2
   % Frames information:
   %         Width : 512 pixel
   %         Height: 512 pixel
   %         Global minimum: 3987.0
   %         Global maximum: 41004.0

   %         Per frame information (verbose output):
   % Frame 0:
   %         57 particles found
   %         Detected particle positions:
   ```

   The procedure to convert this file to a MatLab format is the following: you have to run APM_GUI on MatLab. After the graphical interface appears, you can select the files to convert using the browser for the folder dialog box that appears after you press the "Browse" button in the panel "From ImageJ". You can select more than one file using the keys Ctrl and Shift, as usual. When your files to convert have been selected, press the "Convert" button in the panel "From ImageJ". Information about the conversion status is displayed on MatLab Command Window. New .mat files will be generated and saved in the same folder where the .txt files are located. This application has been tested on Particle Tracker version 1.5.

3. **Using MTT.** After MTT is run and its folder is defined data as the current directory, the script convertMTT2mat has to be run on MatLab. A new .mat file is created and saved in the MTT folder output22, and its filename would be the same as that of the movie you analyzed with MTT followed by _converted.mat. After the script is run convertMTT2mat, the workspace generated by MTT is cleaned. To keep the MTT output for MatLab, its workspace has to be saved before running our script. The MTT folder detect_part_v2 also has to be in the MatLab path, and you can add the file convertMTT2mat to that folder. This application has been tested using MTT version 2.2.

4. **Using any other SPT software.** APM_GUI uses .mat files that can be generated by saving a workspace with variables that are called particle1, particle2, ... particleN and N. Each one of the variables particle$i$, with $i = 1 \dots N$, contains the information for the trajectory number i. Each of these variables is a vector with three columns: the first and second columns have the x and y coordinates of the particle i, and the third column has the frame number. Finally, N is the number of trajectories.

Finding confined regions

To find regions in which the particles have confined movement, run APM_GUI on MatLab. After the graphical interface appears, you can select the files to analyze by using the browser for the folder dialog box that appears after you press the "Browse" button in the panel "Analyzing". The files to analyze must have a .`mat` extension and contain the trajectory information, (these are the files that you have generated using the functions in the panel "Converting files"). You can select more than one file using the keys `Ctrl` and `Shift`, as usual. When your files to analyze have been selected and the required parameters have been given, the analysis will start after you press the "Finding confinement" button in the panel "Analyzing". Information about the analysis status is displayed on MatLab `Command Window`. New .`dat` files and a new .`mat` file will be generated and saved in the same folder where the .`mat` files are located. A detailed explanation of inputs and outputs is given below. To prevent mistakes, all the inputs are deleted after the analysis is complete.

The inputs

All of the parameters needed to analyze the diffusive properties of a particle have to be entered in the empty boxes. An explanation of each parameter is given below, and a summary is given in Table 2.

1. **Time between frames (s).** The time between frames **in seconds** has to be entered in this box.
2. **Minimum diffusion coefficient (d^2/s).** This is the minimal value for the diffusion coefficient that is required to consider the particle as mobile. The analysis does not consider particles with Brownian motion characterized by a diffusion coefficient (taken as the median of the instantaneous diffusion coefficients) less than this value. If a confined region is characterized by a diffusion coefficient less than this value, then the particle is considered to be immobile in that region. The value that you have to introduce here depends on your experimental equipment and is the smallest value you can measure. The brackets represent the unit, where d indicates the unit used for distance and depends on the tracking software. The time always has to be introduced in seconds.
3. **Exposure time.** The camera exposure time has to be entered in this box **in seconds**.
4. **Minimal amount of points.** After tracking, you obtain many trajectories with different lengths. Some of them may not be sufficiently long. Thus, the analysis will take into account only the trajectories that are present in a greater number of frames than the parameter that has to be introduced in this box.
5. **Most wide window.** The parameter S_m has to be introduced in this box. You have to take into account that S_m **cannot be greater than the number introduced in the "Minimal amount of points" box**. We suggest that you select the lasted duration to be approximately twice the value of S_m. Thus, you can be sure that you use at least half of the trajectory points for the analysis.
6. **Alpha.** In this box, you have to introduce the parameter α, which is described in our paper and is one of the most important parameters because it defines L_c. You have to optimize it.
7. **Minimum L_c.** The minimal value for the threshold L_c has to be entered in this box. A particle with random Brownian motion should always have a confinement index below this threshold.
8. **Maximum L value to fix a threshold.** If the confinement index has a value greater than the one entered in this box, the threshold is defined as the value introduced in the "Threshold for high L" text box. This setting is designed to prevent mistakes in the definition of confined region sizes in the cases of trajectories that present confinement most of the time or when there is a large difference between the diffusion coefficients inside and outside of the confined regions.

9. **Threshold for high L.** This is the threshold value, L_c, if at some point in the trajectory, L has a greater value than that entered in the "Maximum L value to fix a threshold" text box. If you do not want to use this value in the analysis, you have to enter a very large value in "Maximum L value to fix a threshold".

10. **Minimal time to consider confinement (s).** This value represents the minimal time required to consider that a particle has a confined movement if its confinement index is above the threshold for at least that period of time. **It has to be expressed in seconds!**

11. **Minimal width or height.** Trajectories located in regions with a width or height less than this value are not considered, which prevents incorrect tracking on the boundaries.

12. **Project's name.** Filling in the "Project's name" text box is optional. Files generated by APM_GUI have default names, but if you introduce a name for the project, for instance *pname*, default names are followed by "_*pname*". We recommend that you create a folder for each different group of data that was obtained using different experimental conditions. Therefore, you will be always sure that all files generated by APM_GUI and located in a single folder belong to the experimental condition correlated with that folder, even if you did not enter text in the "Project's name" text box.

13. **File(s) to analyze.** Select the files to analyze using the browser for the folder dialog box that appears after the "Browse" button is pressed in the panel "Analyzing". You can select more than one file using the keys `Ctrl` and `Shift`, as usual.

Input variable	Default	Notes
Time between frames		Experimental parameter.<br>It must be in seconds.
Minimum diffusion coefficient		Experimental parameter.<br>It depends on the equipment resolution.
Exposure time		Experimental parameter.<br>It must be in seconds.
Minimal amount of points	50	It should be around twice S_m.
Most wide window	25	Critical if too small or too large.
Alpha	0.5	Number between 0 and 1.<br>It should not be near 0.
Minimum L_c	4.3	Should be greater than L for a pure random walk.
Maximum L value to fix a threshold	100	It should not be used in most trajectories.
Threshold for high L	19	It should be considerably greater than Minimum L_c.
Minimal time to consider confinement		It could depend on your sample.<br>It must be in seconds.
Minimal width or height		It prevents mistakes at the boundary.

Table 2. Input variable summary

Other options available in the panel "Analyzing" are referred to as plotting options. If you ask for plotting, the confined regions are represented by light and dark blue circles, which indicate mobile and immobile particles, respectively. Small green and red circles indicate the first and last points of the trajectory, respectively. You can choose to plot all trajectories, trajectories with non-confined regions or trajectories with confined regions. You can also ask to stop the analysis after each plot. In this case,

press any key after the plot appears to continue. An example of the plot is shown in Fig. 2 (see the article).

For the analysis, you can select as many files as you want, which will allow you to analyze many data at the same time, (for example, all of the data obtained for different trials from the same culture). However, when more files are analyzed, more time is required.

The outputs

Five files will be generated after the APM_GUI's analysis is finished. The default names of these files are as follows: `generaldata.dat`, `dataconfinement.dat`, `dataconfinementim.dat`, `dataconfinementoutside.dat` and `workspace.mat`. If you have entered text in the "Project's name" text box, then the file names are followed by *_enteredtext*. The temporal unit is always seconds, but the spatial unit depends on the track. A detailed description of these files is given below.

The file generaldata.dat

This file has general information about all trajectories and has six columns with the following data:

1. The first column has the `.mat` file names.
2. The second column has the trajectory numbers.
3. The third column indicates **Conf** if the trajectories have confined regions and **nonConf** if they do not.
4. The fourth column has the total times for each trajectory.
5. The fifth column has the values of L_c.
6. The last column has the diffusion coefficients for particles without confined regions.

The file dataconfinement.dat

This file has information about trajectories with confined regions of movement for **mobile particles**. **This information is only for confined regions.** It has ten columns with the following data:

1. The first column has the `.mat` file names.
2. The second column has the trajectory numbers.
3. The third column has the durations of the confinements.
4. The fourth column has the characteristic diffusion coefficients inside the confined regions.
5. The fifth column has the radius of the confined regions (a measure of confined region sizes).
6. The sixth column has the x-coordinates for the confined region centers.
7. The seventh column has the y-coordinates for the confined region centers.
8. The eighth column has the threshold values L_c that were used for this trajectory.
9. The ninth column has the frame numbers in which particles start their confined movements.
10. The tenth column has the frame numbers in which the particles finish their confined movements.

The file dataconfinementim.dat

This file has information regarding trajectories with confined regions of movement for **immobile particles**. **This information is only for confined regions that are characterized by diffusion coefficients below the value introduced in the "Minimum diffusion coefficient (dˆ2/s)" text box.** It has nine columns with the following data:

1. The first column has the .mat file names.
2. The second column has the trajectory numbers.
3. The third column has the durations of the confinements.
4. The fourth column has the radius of the confined regions (a measure of the size of the confined region).
5. The fifth column has the x-coordinates for the confined region centers.
6. The sixth column has the y-coordinates for the confined region centers.
7. The seventh column has the threshold values L_c that were used for this trajectory.
8. The eighth column has the frame numbers in which the particles start their confined movements.
9. The ninth column has the frame numbers in which the particles finish their confined movements.

The file dataconfinementoutside.dat

This file has the information for trajectories with confined regions. **This information is only for characteristic diffusion coefficients outside confined regions.** It has three columns with the following data:

1. The first column has the .mat file names.
2. The second column has the trajectory numbers.
3. The third column has the characteristic diffusion coefficients outside the confined regions.

The Workspace

If you are familiar with MatLab, you can use it to analyze the results. All of the relevant data are saved in a .mat file with a default name of workspace.mat. If you have entered text in the box "Project's name", the file name is followed by *_enteredtext*. You can load this file at any time. A list of the variables in the file is given below.

1. *DConf_inside*: diffusion coefficients in confined regions (mobile particles).
2. *DConf_outside*: diffusion coefficients of particles outside the confined regions (only trajectories with confined regions are considered).
3. *DnonConf*: diffusion coefficients considering whole trajectories without confined regions.
4. *FileNameConf_inside*: .mat filenames. The confined region characterized by a diffusion coefficient given by $DConf\_inside(i)$ is in the .mat file with the name $FileNameConf\_inside\{i\}$.
5. *FileNameConf_inside_immobile*: .mat filenames. The confined region characterized by a size given by $R\_immobile(i)$ is in the .mat file with the name $FileNameConf\_inside\_immobile\{i\}$.

6. $FileNameConf\_outside$: .mat filenames. A trajectory with confined regions that is characterized outside these regions by a diffusion coefficient given by $DConf\_outside(i)$ is in the .mat file with the name $FileNameConf\_outside\{i\}$.

7. $FileNameNonConf$: .mat filenames. A trajectory without confined regions, i.e., corresponding to a particle with Brownian motion, and the characterized diffusion coefficient given by $DnonConf(i)$ is in the .mat file with the name $FileNameNonConf\{i\}$.

8. $Final\_time\_Conf\_inside$: number of frames in which mobile particles leave confined regions.

9. $Final\_time\_Conf\_inside\_immobile$: number of frames in which immobile particles leave confined regions.

10. $Initial\_time\_Conf\_inside$: number of frames in which mobile particles arrive at confined regions.

11. $Initial\_time\_Conf\_inside\_immobile$: number of frames in which immobile particles arrive at confined regions.

12. Lc_Conf_inside: L_c for mobile particles with confined regions.

13. $Lc\_Conf\_inside\_immobile$: L_c for immobile particles with confined regions.

14. $Lc\_nonConf$: L_c for trajectories without confined regions.

15. $ParticleNumberConf\_inside$: number of mobile particles with confined regions.

16. $ParticleNumberConf\_inside\_immobile$: number of immobile particles with confined regions.

17. $ParticleNumberConf\_outside$: number of particles for which the diffusion coefficients outside the confined regions could be calculated.

18. $ParticleNumberNonConf$: number of particles without confined regions.

19. R: radius of the confined regions (mobile particles).

20. $R\_immobile$: radius of the confined regions (immobile particles).

21. $Time$: how long particles stay in a confined region in seconds (mobile particles).

22. $Time\_immobile$: how long particles stay in a confined region in seconds (immobile particles).

23. $TotalTimeTrajectoryConf$: trajectory duration in seconds for mobile particles with confined regions.

24. $TotalTimeTrajectoryNonConf$ trajectory duration in seconds for particles without confined regions.

25. $parameters$: the entered numbers (in the empty boxes).

26. xcm: x-coordinate for confined region centers (mobile particles).

27. $xcm\_immobile$: x-coordinate for confined region centers (mobile particles).

28. ycm: y-coordinate for confined region centers (mobile particles).

29. $ycm\_immobile$: y-coordinate for confined region centers (immobile particles).

Exporting a trajectory

Using the panel “Exporting a trajectory”, it is possible to export the x and y coordinates in a .dat file, along with the confinement index L and the instantaneous diffusion coefficient over time. Select the file that contains the trajectory that you wish to export. For this step, you have to use the browser for the folder dialog box that appears after you press the “Browse” button in the panel “Exporting a trajectory”. You can select only one file and you have to enter the number of the trajectory that you wish to export as well as the parameters required in the empty boxes in the panel “Analyzing”. The goal of this application is to export the functions that are plotted during the analysis for a particular trajectory. The number of the trajectory is given at the top of each plot or in the exported files. After the Export trajectory button is pressed, a new file called filename-trajectory_number.dat is created and saved in the same folder where the selected file is located. It has five columns. The first and second columns have the x and y coordinates, the third column has the numbers of frames, the fourth column has the confinement indices, and the fifth column has the instantaneous diffusion coefficients. Information about the exportation status is displayed on the MatLab Command Window.

Note: If you wish to save a plot from the analysis, first select the MatLab window of the plot that you want. Then, you have to write in the MatLab Command Window: print(gcf,'-djpeg','-zbuffer','-r300','namefileplot.jpg') or print(gcf,'-depsc','-zbuffer','-r600','namefileplot.eps') and press Enter. The first option would create a .jpg file called namefileplot.jpg at 300dpi in the MatLab Current Directory. The second option would create a .eps file called namefileplot.eps at 600dpi in the MatLab Current Directory.

Example

In the folder Example, you have small test files for APM_GUI. For the files located in the folder ToAnalyze, we use the following parameters: *Time between frames (s)*, 0.067; *Minimum diffusion coefficient (d^2/s)*, 0.0075; *Exposure time*, 0.037; *Minimal amount of points*, 50; *Most wide window*, 25; *Alpha*, 0.5; *Minimum L_c*, 4.3; *Minimal time to consider confinement (s)*, 0.67; *Maximum L value to fix a threshold*, 100; *Threshold for high L*, 19; *Minimal width or height*, 0.48. The other folders have small samples to convert using From Imaris or From ImageJ. We do not provide an example in MTT because a previous installation of MTT is required to convert the files.
