# Supplementary material for: APM_GUI: analyzing particle movement on the cell membrane and determining confinement
Source: BMC Biophys. 2012 Feb 20;5:4. doi: 10.1186/2046-1682-5-4 (PMC3337278; doi:10.1186/2046-1682-5-4)
Supplement: Additional file 2 — Materials and Methods. This file has a description of the materials and methods for the biological samples that were used to test our software. Hippocampal neurons from 18- day- old rat embryos were cultured until 10-14 DIV on glass coverslips, according to the Banker technique [19] density of (~ 6000 neurons/cm2). The protocol described in Ref. [20] was used to label GluR2-containing AMPARs. [file 2046-1682-5-4-S2.PDF]

Materials and Methods

Cell culture and reagents

Hippocampal neurons from 18-day-old rat embryos were cultured until 10-14 DIV on glass coverslips, according to the Banker technique [19] density of ($\sim$ 6000 neurons/cm^2). The neurons were maintained on a glial feeding layer under 5% CO_2 at 37°C in minimal essential medium with N2 supplement (MEM-N2).

GluR2 live cell staining for single-particle tracking

The protocol described in Ref. [20] was used to label GluR2-containing AMPARs. The neurons were incubated for 10 min at 20°C in mAb primary antibody (2.5 μg/ml in N2 medium supplemented with 20mM HEPES; BD Pharmingen), washed and incubated for 10 min at 20°C in biotinylated anti-mouse Fab antibody (2 μg/ml in N2 medium supplemented with 20 mM HEPES, Fab/Biotin ratio $\sim$1:0.6). Following the washes, the coverslips were incubated for 1 min at RT in streptavidin-coated QDs with emission at 709 nm (0.2-0.7 nM Invitrogen) in borate buffer (50 mM) supplemented with sucrose (100 mM). After quick rinses, the coverslips were mounted in a custom chamber with preincubated Modified Hanks medium. The Fab antibody (AffiniPure Fab Fragment, Jackson Immuno Research) was biotinylated using the Biotin-XX Microscale Protein Labeling Kit (Invitrogen). All washes and incubations were performed in fresh N2 medium supplemented with HEPES (20 mM).
Glutamate was added to the recording medium at a final concentration of 100μm.

Single-molecule optical microscopy

The cells were imaged at 37°C in a closed chamber mounted on an inverted microscope (Olympus, IXL-UCB) equipped with a 100X objective (NA=1.45, Olympus). The fluorescent images of the QDs were obtained with an integration time of 64 ms using a CCD camera (Hamamatsu), with up to 1000 consecutive frames acquired with CellR (Universal Imaging).

Receptor tracking

Single-molecule tracking was performed with the Imaris program. The center of the fluorescence spots was determined with a Gaussian fit at a spatial resolution of 10 nm. Single QDs were identified by the fluorescence intermittency, i.e., the alternation of periods when the QD emitted fluorescent photons and periods when it was dark. Because the observations were based on random blinking events, the trajectory of individual receptors could not be tracked continuously. Thus, different parts of the trajectory were attributed to the same receptor only when the positions of the spot before and after the dark period were compatible with the duration of the extinction and the typical diffusion coefficient.
